# Addison's Disease

**Published:** 1890-05-10

**Authors:** 


### THE PRACTITIONER'S MIRROR AND RETROSPECT.

[*The Editor will be glad to receive offers of co-operation and contributions from members of the profession. There is no doubt that much valuable material full of interest and instruction is at present lost to science. This is largely so in the case of* (1) *the younger and less-known members of the hospital staff* (2) *those connected with cottage hospitals, and* (3) *the great body of medical practitioners not attached to any institution. The co-operation of all is cordially invited to enable the Editor to make* THE HOSPITAL *of the utmost possible use and value to the profession. Specialists willing to review books on their special subjects are invited to communicate this fact at once. All letters should be addressed to* THE EDITOR, THE LODGE, PORCHESTER SQUARE, LONDON, W.]

### MEDICINE.

### ADDISON'S DISEASE.

A clinical lecture by Professor H. Nothnagel, Professor of Medicine in the University of Vienna, appears in the *Medical Press and Circular* on "Morbus Addisonii." Addison described the disease under the group of grave anæmias. But Professor Nothnagel asserts this is not correct, for there are patients in which there is no trace of either oligocythæmia or oligochromæmia. There are cases in which the tongue and lips retain the natural red colour. But, says the lecturer, there is an adynamia, that has nothing to do with anæmia. Adynamia is synonymous with nervous prostration, and is a fundamental symptom of Addison's disease. Generally the bronzing progresses simultaneously with the adynamia. The essential symptoms are their simultaneous appearance, on which "you may conclude a case to be one of Addison's disease." Bronzing alone, even when the oral mucus membrane is implicated, is not sufficient for diagnosis. Disturbance of the digestive tract always appears eventually. The prognosis is a lethal one. How death occurs varies. It may be from prostration, or from profuse evacuations. There are also cases that end in coma; in others auto-intoxication takes place. Pathological anatomy teaches that, in the event of the occurrence of a group of symptoms, the supra-renal capsules are often found caseated. But it has been observed that other diseases of the capsules may lead to the symptoms of Addison's disease, and, amongst others, carcinoma. But, still further, cases have exhibited the typical symptoms of Addison's disease, and at the necropsy the supra-renal capsules were found normal. Thus, in one case degeneration of the pancreas was discovered, whilst the capsules were unchanged. But we have no right to attribute Addison's disease to degeneration of the pancreas. The essential condition is believed by Professor Nothnagel to be affection of the nerves, of the sympathetic, of the solar plexus, or of the splanchnic, &c. The essential is lesion of the nerves, and especially of the abdominal nerves. The chief aim of the physician is to keep up the strength by iron, wine, good food, &c. Æthereal tincture of the acetate of iron, or the so-called Bestnscheff's nervino-tonic mixture is recommended.

Now, on turning to the original memoir of Dr. Addison we find that he wrote as follows: "The leading and characteristic features of the morbid state to which I would direct attention are anæmia, general languor, and debility, remarkable feebleness of the heart's action, irritability of the stomach, and a peculiar change of colour of the skin, occurring in connection with a diseased condition of the supra-renal capsules." It was soon discovered, however, that the general symptoms of the disease, as given above, outweigh in importance any pigmentary change. It was also soon discovered that such symptoms may occur (as pointed out by the Vienna professor) without alteration of the supra-renal capsules being found. Briefly, it may be said that when changes occur in the supra-renal capsules they consist of thickening of the connective tissue surrounding the nerve fibres, and the ganglion cells, giving rise to compression and ultimate destruction of nerve cells and fibres. Similar changes have also been noted in the cerebro-spinal nerve fibres and in those more intimately connected with the ganglionic system. Dr. Silver long since remarked: "The exact mode in which these nerve lesions give rise to the characteristic symptoms of the disease are as yet matters of speculation, and not of exact science." The Vienna professor, therefore, cannot claim any originality in the matter, neither does his clinical lecture add much to our knowledge as to the cause of the disease. One fact, however, stands prominent. Addison's disease is in many instances associated with an hereditary constitution of the tubercular or scrofulous type. Notwithstanding Professor Nothnagel's theory of the origin of the malady, we must still regard Addison's disease as one of the multitudinous forms of anæmia. Any variety of anæmia may be as satisfactorily traced back to the nerves as Addison's disease. Anæmia depends upon blood degeneration, and this blood degeneration is associated with many presumed causes. Whether this blood degeneration initiates in the blood itself, or whether subtle changes occur in the nervous system previously, must be regarded as *sub judice*.

